# Sexual dysfunction and its associated factors among patients with heart failure in public hospitals in Gondar town, North and West Gondar, 2024

**DOI:** 10.1186/s12978-024-01883-1

**Published:** 2024-10-16

**Authors:** Mihret Melese, Mequanent Tiruneh Tassew, Gizachew Kassahun Bizuneh

**Affiliations:** 1 Department of Human Physiology, School of Medicine, College of Medicine and Health Science, University of Gondar, Gondar, Ethiopia; 2 Department of Surgery, School of Medicine, College of Medicine and Health Science, University of Gondar, Gondar, Ethiopia; 3 Department of Pharmacognosy, School of Pharmacy, College of Medicine and Health Science, University of Gondar, Gondar, Ethiopia

**Keywords:** Sexual dysfunction, Hear failure, Prevalence, Associated factors, Ethiopia

## Abstract

**Introduction:**

Sexual dysfunction is characterized by difficulties that interfere with achieving satisfying sexual activity, affecting desire, arousal, orgasm, and overall satisfaction. A recent study in Ethiopia identified heart failure as one of the most common cardiovascular diseases in the country. Sexual dysfunction significantly affects the quality of life in individuals with heart failure, yet it often goes underdiagnosed and underreported. Understanding the prevalence of sexual dysfunction and the factors influencing sexuality in these patients is essential. Therefore, the primary objective of this study was to determine the prevalence of sexual dysfunction among patients with heart failure in Gondar City and the northern and western zones of Gondar public hospitals.

**Method:**

This study included 423 participants diagnosed with heart failure, selected through simple random sampling from January 3 to February 30, 2024. Data were collected using an interviewer-administered questionnaire covering socio-demographic, behavioral, and clinical information. Sexual dysfunction in males was assessed using the International Index of Erectile Function-5, while the Female Sexual Function Index-6 was used for females. Data were entered into Epidata version 3.6 and later exported to SPSS version 25 for analysis. Binary logistic regression was employed to identify factors associated with sexual dysfunction, with a significance level set at p ≤ 0.05.

**Result:**

A total of 423 heart failure patients participated in the study, achieving a 100% response rate. The results indicated a significant prevalence of sexual dysfunction, affecting 57.92% of participants (95% CI 54.76%–63.76%). Female participants reported a higher prevalence of sexual dysfunction, with 63% of the 138 female heart failure patients affected. Among the 285 male participants, 55.4% (158 patients) were identified as having erectile dysfunction. Multivariable logistic regression analysis revealed that factors such as body mass index, age, insufficient physical activity, and smoking were significantly associated with sexual dysfunction in females. In males, erectile dysfunction was significantly linked to diabetes mellitus, heart failure classification, age, and insufficient physical activity.

**Conclusion and recommendation:**

This study identified a high prevalence of sexual dysfunction, with females being more affected than males. Additionally, the research identified several factors influencing sexual dysfunction among patients with heart failure, including BMI, age, cigarette smoking, diabetes mellitus, and the classification of heart failure. The study recommends that healthcare providers and other stakeholders take proactive measures to alleviate the burden of sexual dysfunction in patients with heart failure. Strategies should focus on controlling the severity of heart failure symptoms, effectively managing comorbidities, and addressing factors such as body weight, psychological well-being, and behavioral patterns. By targeting these areas, healthcare providers can work toward minimizing the risk of sexual dysfunction and improving the overall quality of life for patients with heart failure.

## Introduction

Sexuality, a fundamental aspect of human life encompasses sex, sexual orientation, eroticism, pleasure, intimacy, and reproduction. Sexual health involves more than just the absence of disease or dysfunction; it includes physical, emotional, mental, and social well-being in relation to sexuality [1]. Sexual dysfunction is defined as the presence of difficulties that hinder the achievement of satisfying sexual activity, affecting various aspects, including desire, arousal, orgasm, and overall satisfaction [2–4]. Men may experience erectile dysfunction, premature or delayed ejaculation, or low libido, whereas women may face challenges such as difficulty in arousal, achieving orgasm, experiencing painful intercourse, or dealing with veganism [5–8].

Approximately 60 to 87% of heart failure (HF) patients report sexual problems, with only 31% of those younger than 70 maintaining normal sexual function. Studies indicate that women with HF experience a wider range of sexual difficulties compared to men. For example, in the general population, 27% of women report a lack of interest in sexual activity, while 23% struggle to achieve orgasm. In contrast, men with HF report significantly higher rates of erectile dysfunction (ED) [9–11]. However, many still value their sexual health; a study by Alberti et al. found that 52% of men and 38% of women with HF consider sex an important aspect of their lives, significantly affecting their overall quality of life. Supporting this, research by Steinke et al. revealed that 80% of female HF patients experience reduced vaginal lubrication, and 76% report frequent unsuccessful intercourse [11, 12]. In a study conducted in Iran, 80% of men with heart failure reported experiencing sexual dysfunction, with 36% classified as severe, 26% as moderate, and 18% as mild. Research on this issue in Africa is limited [13]. A study in Cameroon found a prevalence of 57.7% and identified three main disorders: issues with sexual desire, difficulties with vaginal lubrication, and erectile dysfunction. Risk factors included being female, being over 60 years old, beta-blocker use, hypertension, and fear of experiencing a heart attack during [14].

Heart failure (HF) affects physical function, affecting both patients and their partners’ daily lives. Patients may experience decreased sexual performance, satisfaction, and interest as well as a reduction in the frequency of sexual activity [14, 15]. The global burden of diseases estimated that over 64.3 million people worldwide are affected by heart failure, with its epidemiology varying both within and between countries and continents [14]. Sexual dysfunction among patients with heart failure may be attributed to several factors. Heart failure can directly reduce blood flow to the genital area, affecting erectile function in men and arousal in women. In addition, the emotional toll of living with heart failure, such as anxiety, depression, or stress, can also contribute to sexual dysfunction. Moreover, heart failure and its treatment may disrupt hormonal balance, further affecting sexual function [16–19].

According to a population-based cohort study conducted in the UK, the odds of survival following a heart failure diagnosis have improved somewhat in the twenty-first century, but they are still lower than those of other serious illnesses like cancer [20]. Hospital-based studies show that heart failure is the most common primary diagnosis among patients admitted with cardiovascular disease, accounting for 9–15% of hospital admissions, although comparable estimates are not currently available for Sub-Saharan Africa (SSA) [21]. In the SSA, survival rates are especially low, with six-month mortality rates estimated to be close to 20% [21–23].

A recent study conducted in Ethiopia revealed that Heart Failure (HF) is one of the most prevalent cardiovascular diseases in Ethiopia, affecting a significant 23.9% of the population [24]. Sexual dysfunction significantly impacts the quality of life of individuals with heart failure, yet it remains underdiagnosed and underreported. It is crucial to determine the magnitude of sexual dysfunction and address the factors affecting their sexuality. Hence, the primary objective of this study was to determine the prevalence of sexual dysfunction among patients with heart failure in Gondar City and the northern and western zones of Gondar public hospitals. Ultimately, the results of this study will have important implications for healthcare providers, policymakers, and patients, helping to shape more effective and patient-centered approaches to managing heart failure and promoting healthy sexual function.

## Materials and methods

### Study design and settings

An institutional cross-sectional study was conducted from January 3 to February 30, 2024, at seven public hospitals across north Gondar, west Gondar, and Gondar City. Located in the northwestern part of Ethiopia within the Amhara Regional State, Gondar is situated at 12°3′N latitude and 37°28′E longitude. The city lies 727 km from Addis Ababa, the federal capital, and 120 km from Bahir Dar, the capital of the Amhara National Regional State. Covering a total area of 192.3 km^2^, Gondar is characterized by undulating mountainous terrain. According to the 2007 National Population and Housing Census, Gondar comprises 50,817 housing units and serves as the political and economic hub of the North Amhara region, being the main city of the North Gondar Zone. The city is divided into 12 administrative sub-cities, each with its own legislative, executive, and judiciary branches. Gondar City has two public hospitals (University of Gondar Specialized Hospital and Ayra General Hospital). Debark town, the capital of the North Gondar Zone, is located 755 km from Addis Ababa and hosts three public hospitals (Debark General Hospital, Janitor Primary Hospital, and Dabat Primary Hospital). The capital of the West Gondar Zone, Genda Wuhan, situated 790 km from Addis Ababa, has two public hospitals (Metema General Hospital and Abrajira Hospital). The total number of adult heart failure patients receiving follow-up care at the University of Gondar Specialized Hospital and Ayra General Hospital was 246. In the North Gondar hospitals, there were 294 heart failure patients, while the West Gondar public hospitals had 214 heart failure patients attending follow-up care. The study included patients aged 18 years or older with a previous diagnosis of heart failure who had at least one month of follow-up prior to the data collection period.

### Population and eligibility criteria

The population source included all heart failure patients attending seven public hospitals in Gondar City, West Gondar, and North Gondar. The study population consisted of patients with heart failure who met the inclusion criteria.

### Inclusion criteria

This study included all patients with heart failure aged > 18 years who attended the seven public hospitals during the data collection period.

### Exclusions criteria

Heart failure patients who were critically ill or had mental disabilities were excluded from the study.

### Sampling size determination

Sample size was determined using a single population proportion formula by considering assumptions of the proportion of parent-adolescent communicating on sexual and reproductive health issues assumed to be 50% (i.e. p = 0.5), 95% confidence level, and a 5% margin of error.

$$\begin{aligned} {\text{ni}} = & \frac{{({\text{Z}}\alpha /{2})^{{2}} *{\text{P }}\left( {{1} - {\text{P}}} \right)}}{{{\text{d}}^{{2}} }} \\ = & \frac{{({1}.{96})^{{2}} 0.{5 }\left( {{1} - 0.{5}} \right)*{2}}}{{{\text{d}}^{{2}} }} = \frac{{{3}.{8416}*0.{25}}}{{\left( {0.0{5}} \right)^{{2}} }} = \frac{{0.{96}0{4}}}{{0.00{25}}} = {384}.{16} \\ \end{aligned}$$

Finally, considering a 10% non-response rate, 384.16 + 38.416 = 422.576 ≈ 423,

Finally, with a 10% non-response rate, the minimum calculated sample size was 423.

### Sampling technique and procedure

**Fig. 1:**
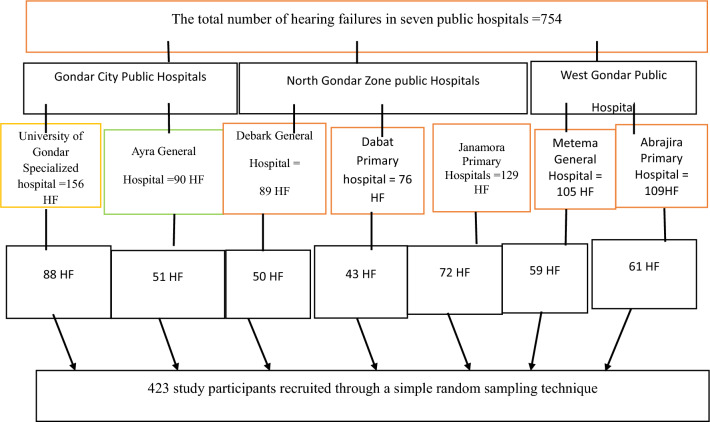
Sampling procedure to determine the prevalence of sexual dysfunction-associated factors among heart failure patients in Gondar town, west Gondar, and north Gondar public hospitals, 2024(n = 423)

The study randomly selected seven public hospitals from the Gondar region using a lottery method. A total of 423 participants with heart failure were recruited through simple random sampling from these hospitals: University of Gondar Specialized Hospital, Ayra General Hospital, Debark General Hospital, Janamora Primary Hospital, Dabat Primary Hospital, Metema General Hospital, and Abrahajia Hospital. Participants were allocated proportionately based on each hospital's size and capacity to ensure a representative sample. Data collection was conducted at each hospital (Fig. 1).

### Study variables

*Dependent variable*: Sexual dysfunction was defined using the International IIEF-5 for men, with a score of 22 or below indicating erectile dysfunction, and FSFI-6 for women, with a score below 26 indicating sexual dysfunction.

*Independent variables*: This study investigated socio-demographic, clinical, and behavioral factors of sexual dysfunction. Socio-demographic factors included age, marital status, residence, education, BMI, and occupation. Clinical factors included heart failure severity, duration, medications, and comorbidities like diabetes, stroke, chronic kidney disease, and depression. Behavioral factors include physical activity, alcohol use, cigarette smoking, and khat chewing. These variables offer insights into hypertension and its health consequences.

### Operational definition

*Sexual dysfunction*: In this study, sexual dysfunction in men was assessed using the International Index of Erectile Function-5 (IIEF-5), with a score of 21 or below indicating erectile dysfunction (ED) [25, 26]. For women, sexual dysfunction was evaluated using the Female Sexual Function Index-6 (FSFI-6), where a score below 26 indicated dysfunction [27].

*Heart failure*: Heart failure was defined as all adult patients with a diagnosis of HF who met the Framingham criteria retrospectively and had been under follow-up at a specialized cardiology clinic for at least 3 months. To confirm the diagnosis of heart failure, documented evidence of either two major criteria or one major and two minor criteria not attributable to another medical condition was required [28, 29].

The Framingham Diagnostic Criteria are as follows:

*Major Criteria* Acute pulmonary edema, cardiomegaly, hepatojugular reflex, pulmonary rales, third heart sound (S3 gallop), weight loss of 4.5 kg or more in response to treatment, neck vein distension, and paroxysmal nocturnal dyspnea or orthopnea were the major criteria [30].

*Minor Criteria*: Minor criteria included ankle edema, hepatomegaly, nocturnal cough, pleural effusion, dyspnea with exertion, and tachycardia (defined as heart rate exceeding 120 beats per minute) [30].

*Hypertension*: It was defined according to the eighth Joint National Committee guideline (JNC-8). Stage I hypertension was identified as a systolic blood pressure (SBP) of 140–159 mmHg or a diastolic blood pressure (DBP) of 90–99 mmHg, whereas stage II hypertension was indicated by an SBP of 160 mmHg or higher or a DBP of 100 mmHg or higher in male hypertensive patients using antihypertensive medications. Controlled hypertension was defined as an SBP/DBP of 140/90 mmHg. Blood pressure measurements were taken twice in a seated position using a standard Mercury sphygmomanometer (Mercurial, India) with a cuff size that covered at least two-thirds of the upper arm. Participants were required to rest for at least 5 min and refrain from smoking and consuming caffeinated beverages for 30 min before measurement. The average of two readings taken 5 min apart was recorded as the final blood pressure reading. The staging of hypertension was categorized according to the JNC-8 guidelines [31].

*Body Mass Index (BMI)*: In this study, participants with a Body Mass Index (BMI) of 25 kg/m^2^ or higher were classified as overweight [32]. The participants’ weight was measured while they wore light clothing and stood barefoot and was recorded in kilograms. Height was measured to the nearest 0.5 cm using stadiometer. To ensure accuracy, the weight measuring scale was checked and reset to zero between each measurement. The Body Mass Index (BMI) of the participants was calculated by dividing their weight in kilograms by the square of their height in meters. The resulting BMI values were then categorized as underweight (< 18.5 kg/m^2^), normal weight (18.5–24.9 kg/m^2^), or overweight (≥ 25 kg/m^2^) [32].

*Diabetic mellitus*. Good glycemic control is characterized by fasting blood glucose levels between 70 and 126 mg/dl. On the other hand, poor glycemic control occurs when fasting blood glucose levels are either below 70 mg/dl or exceed 126 mg/dl [33].

*Depression*: Individuals who scored 10 or higher out of a possible 27 points on the Patient Health Questionnaire-9 (PHQ-9) were diagnosed with depression, whereas those who scored between 0 and 9 were classified as not having depression [34]. Depressive symptoms were assessed using PHQ-9, a standardized tool that evaluates the presence and severity of depression over the 2 weeks leading up to the study. According to the PHQ-9 scoring criteria, participants with a score of 0–9 were categorized as not experiencing depression, whereas those with a score of 10 or higher were classified as having depression [35].

### Physical activity

#### Use of the International Physical Activity Questionnaire (IPAQ)

Participants will complete the IPAQ to report their physical activity levels over the past week. The IPAQ assesses various types of activities, including Walking, moderate-intensity activities, and vigorous-intensity activities [36].

### Based on IPAQ results, classify participants into categories

*Low Physical Activity*: Individuals who do not meet the criteria for moderate or vigorous activity.

*Moderate Physical Activity*: Individuals who engage in at least 150 min of moderate-intensity activity per week or 75 min of vigorous-intensity activity.

*High Physical Activity*: Individuals who meet or exceed 300 min of moderate-intensity activity per week or 150 min of vigorous-intensity activity [35].

*Ever alcohol drinker*: A heart patient who has consumed at least 12 drinks in their lifetime [37].

*Current alcohol drinkers*: In the last 30 days, heart patients consumed three or more standard drinks per week [37].

*Former cigarette smokers*: A former smoker who has a history of smoking at least 100 cigarettes in his or her lifetime but who has successfully quit smoking and remained smoke-free at the time of the interview [38].

*Current smoker*: An individual who has a lifetime history of smoking at least 100 cigarettes and has smoked a cigarette in the past 30 days [38].

### Data collection tool and procedure

Data were collected by two BSc nurse professionals with experience in data collection of erectile dysfunctions among patients of heart failures. They have been given two days of training by the investigator. Data were collected using a pretested semi-structured questionnaire developed on the basis of a review of relevant literature. Information from the selected participants was gathered through face-to-face interviews and medical chart reviews. The questionnaire was initially prepared in English and then translated into Amharic. To ensure consistency, the manuscript was subsequently translated back into English by language experts.The questionnaire included socio-demographic variables (age, sex, religion, educational background, and occupational status), behavioral variables (physical activity, smoking status, alcohol consumption), and medical-related variables (depression, diabetes mellitus, hypertension, stroke, chronic kidney disease, classification of heart failure, and types of heart failure medication).

The assessment tools also included the IIEF-5) that is used to assess erectile dysfunction among individuals with heart failure. Items are rated on a 5-point likert scale, ranging from 1 to 5. The total scores ranged from 5 to 25, with higher scores indicating the absence of ED. The optimal cut-off score is 22; men scoring 22 or below are categorized as having ED, whereas those scoring above 22 are considered not to have ED [25]. For assessing female sexual dysfunction (FSD), the FSFI-6 is a concise and straightforward measure derived from the original FSFI tool, containing six of its 19 items. It is validated both internationally and locally. Items related to desire and satisfaction is rated on a 5-point likert scale (1 to 5), while the remaining items are rated on a 6-point Likert scale (0 to 5). The total score ranges from 2 to 30, with lower scores indicating poorer sexual functioning. A score below 26 indicates sexual dysfunction. This scale is particularly useful in situations with limited time, such as in this study [27].

### Data management and statistical analysis

Epidata version 3.6 was used for data entry, and the data were subsequently exported to SPSS version 25. The data were checked for completeness. Summary statistics, including proportions and frequencies, were used to summarize the results, which are presented in tables and graphs. A binary logistic regression model was employed to identify the factors associated with dementia. Variables with a p ≤ 0.2 in the bivariable logistic regression were included in a multivariate logistic regression model for adjustment. Crude and adjusted odds ratios (ORs) with 95% confidence intervals (CIs) were calculated. The strength of association was determined using the adjusted OR, with statistical significance set at p ≤ 0.05 in the multivariate binary logistic regression. The Shapiro–Wilk test was used to assess the normality of continuous data. The Homer Lemshow goodness-of-fit test was employed to evaluate the model’s fit. Cornbrash’s alpha was used to assess the validity of the ED questionnaire, resulting in a satisfactory reliability coefficient of 0.88.

### Ethical consideration

The study protocol was approved by the ethical review committee of the School of Medicine, College of Medicine, and Health Sciences, University of Gondar (Ref. No. 567/2023). Written and verbal informed consent was obtained from each study participant after the significance of the study was explained. Potential identifiers were excluded from the questionnaire to ensure confidentiality, and the data were collected in a secure place. All methods were performed in accordance with the relevant guidelines and regulations of the Declaration of Helsinki [39].

## Result

### Background characteristics of the study participants

In this study, 423 participants were included using a simple random sampling technique, which resulted in a 100% response rate. Among these participants, a significant majority (67.4%) were male. The average age of the participants was 36 (1.43 SD) years. The marital status data revealed that most participants were married. Employment status showed that nearly half of the participants were employed either in private sector jobs or in government positions. Educational background analysis indicated that more than 50% of the participants had completed secondary education. Regarding religious affiliation, the data showed that most participants (63.8%) were followers of the Orthodox faith (Table 1).

**Table 1:** Socio-demographic characteristics of study participants in Gondar City, north Gondar, and West Gondar public hospitals, Ethiopia, 2024 (n = 423)

Variables	Category	Frequency (N)	Percentage (%)
Sex	Male	285	67.4
	Female	138	32.6
Age (years)	25–40	129	30.5
	41–60	150	35.5
	> 60	144	34
Marital status	Married	280	66.2
	Single	80	18.9
	Divorced/windowed	61	14.9
Employment	Employed	202	47.7
	Unemployed	128	30.3
	Retired	93	22
Educational status	Primary school (Grade 1th–8th)	106	25
	Secondary school (Grade 9th–12th)	247	58.4
	Tertiary (Diploma and above)	70	16.5
Religion	Orthodox	270	63.8
	Muslim	110	26
	Protestant or catholic	43	10.2
BMI	Normal	193	45.6
	Overweight/obesity	230	54.4

BMI: body mass index

### Clinical and behavioral characteristics of the study participants

Among all patients diagnosed with heart failure, more than 50% had comorbidities, specifically, diabetes mellitus and hypertension. One-quarter of the patients diagnosed with heart failure were found to have chronic kidney disease. More than one-third of the study participants had been diagnosed with heart failure for a duration exceeding 5 years. Additionally, it was found that 30% of heart failure cases were diagnosed at stage NYHA IV. Approximately 17% of individuals with heart failure reported having more than five sexual encounters in the past four weeks (Table 2).

**Table 2:** clinical profile of heart failure in Gondar city, encompassing north and west Gondar Public Hospitals in 2024

Variables	Category	Frequency	Percentage
Diabetic mellitus	Yes	230	54.4
	No	193	45.6
Hypertension	Yes	220	52
	No	203	48
Chronic kidney disease	Yes	105	25
	No	318	75
History of the stock	Yes	147	35
	No	276	65
Duration of heart failure	Less than 1 year	103	24.3
	1–5 years	160	37.8
	> 5 years	160	37.8
Classification of heart failure (NYHA class)	N NYHA I	109	25.8
	YHA II	90	21.3
	NYHA III	95	22.5
	NYHA IV	127	30
Number of sexual encounters in the last 4 weeks	None	178	42
	1–2	83	19.7
	3–4	89	21
	> 5	73	17.3
Smoking	Yes	225	53.2
	No	198	46.8
Alcohol drinking	Yes	245	58
	No	178	42
Physical exercises	Sufficient physical exercise	210	49.6
	Insufficient physical exercise	213	50.4

Hosmer–Lemeshow test = 0.66

BMI: body mass index

*p ≤ 0.05

### Prevalence of sexual dysfunction among hear failure

A total of 423 heart failure patients participated in the study, achieving a 100% response rate. The results indicated a significant prevalence of sexual dysfunction, affecting 57.92% of participants (95% CI 54.76%–63.76%). Female participants reported a higher prevalence of sexual dysfunction, with 63% of the 138 female heart failure patients affected. Among the 285 male participants, 55.4% (158 patients) were identified as having erectile dysfunction.

### Factors associated with sexual dysfunction among female heart failure patients

In the bivariable analysis, factors such as BMI, age, physical activity, cigarette smoking, alcohol consumption, depression, and diabetes mellitus were identified as candidate variables for multivariable logistic regression at a p-value of ≤ 0.2.

However, in the final model, sexual dysfunction among female heart failure patients was significantly associated with BMI, age > 60 years, insufficient physical activity, and smoking, with a p-value of ≤ 0.05. The odds of having sexual dysfunction were higher among overweight or obese female heart failure patients compared to their counterparts (AOR = 1.42; 95% CI 1.09–3.34). Sexual dysfunction was more common in female participants above the age of 60 years than in those between the ages of 25 and 40 (AOR = 3.20; 95% CI 1.08–8.46). Female study participants with insufficient physical activity had 1.6 times higher odds of experiencing sexual dysfunction compared to those with sufficient physical activity (AOR = 1.60; 95% CI 1.19–4.44). Finally, being a cigarette smoker increased the likelihood of experiencing sexual dysfunction compared to non-smokers (AOR = 2.40; 95% CI 1.18–7.66) (Table 3).

**Table 3:** Factors associated with sexual dysfunction among female heart failure patients in Gondar town, North and West Gondar Zone public hospitals, 2024 (n = 138**)**

Variables	Category	Sexual dysfunction		COR	AOR 95%
		Yes	No		
BMI	Normal	40 (67%)	18 (33%)	1	1
	Overweight or obese	37 (53%)	33 (47%)	1.98 (1.13–5.67)	1.42 (1.09–3.34)*
Age (years)	25–40	27 (64.3%)	15 (35.7%)	1	1
	40–60	20 (55.5%)	16 (45.5%)	4.34 (1.17–8.89	0.87 (0.14–3.78)
	> 60	40 (66.7%)	20 (33.3%)	6.82 (1.23–10.45)	3.20 (1.08–8.46)*
Physical activities	Sufficient physical activity	45 (77.6%)	13 (22.4%)	1	1
	Insufficient Physical activity	42 (52.5%)	38 (47.5%)	3.13 (1.12–6.78)	1.60 (1.19–4.44)*
Cigarette smoking	Smoker	51 (47.4%)	11 (52.6%)	5.15 (1.12–8.56)	2.40 (1.18–7.66)*
	None-smoker	36 (34.2%)	40 (65.8%)	1	1
Alcohol drinking	Drinker	28 (47.5%)	31 (52.5%)	0.30 (0.34–0.83	0.24 (0.23–4.60)
	Non-drinker	59 (74.7%)	20 (25.3%)	1	1
Depression	Yes	47 (70%)	20 (30%)	2.99 (1.67–7.98)	1.40 (0.12–5.56)
	No	40 (44%)	51 (56%)	1	1
Diabetic mellitus	Yes	30 (54.5%)	25 (45.5%)	0.55 (0.12–0.78)	0.32 (0.12–2.30)
	No	57 (68.7%)	26 (31.3%)	1	1

Hosmer–Lemeshow test = 0.45

BMI: body mass index

*p ≤ 0.05

### Factors associated with erectile dysfunction in male heart failure patients

BMI, age, physical activity, alcohol consumption, smoking, diabetes mellitus, calcification of heart failure, and categorization of heart failure were among the candidate variables for multivariable logistic regression analysis with a p-value of ≤ 0.02. However, in the multivariable logistic regression analysis diabetic mellitus, classification of heart failure, age (in years), and physical activity were significantly associated with erectile dysfunction at a p-value of ≤ 0.05. The odds of erectile dysfunction among male heart failure patients classified as NYHA IV and NYHA III were higher compared to those classified as NYHA I, with an AOR of 4.67 (95% CI 1.12–8.45) for NYHA IV and an AOR of 3.60 (95% CI 1.09–6.78) for NYHA III. Male study participants aged 40 to 60 years and those over 60 years were more likely to develop erectile dysfunction compared to their counterparts, with an AOR of 4.60 (95% CI 1.13–8.44) for both age groups. Study participants with insufficient physical activity had higher odds of erectile dysfunction compared to those who engaged in sufficient physical activity (AOR = 1.40; 95% CI 1.12–3.55) (Table 4).

**Table 4:** Factors associated with erectile dysfunction among male heart failure patients in Gondar town, North and West Gondar Zone public hospitals, 2024 (n = 285**)**

Variables	Category	Sexual dysfunction		COR	AOR 95%
BMI	Normal	60 (66.7%)	30 (33.5%)	1	1
	Overweight or obese	98 (50.2%)	97 (49.8%)	1.98 (1.07–3.03)	1.40 (0.89–3.44)
Age (years)	25–40	30 (37.5%)	50 (62.5%)	1	1
	40–60	40 (50%)	40 (50%)	4.62 (1.23–9.67)	2.62 (1.08–5.66)*
	> 60	88 (70.4%)	37 (39.6%)	6.62 (1.18–12.55)	4.60 (1.13–8.44)*
Physical activities	In Sufficient physical activity	98 (54.5%)	50 (55.5%)	2.51 (1.12–6.74)	1.40 (1.12–3.55)*
	Sufficient Physical activity	60 (56%)	77 (44%)	1	1
Cigarette smoking	Smoker	100 (62.5%)	60 (36.5%)	1.92 (1.15–7.43	1.62 (0.56–3.66)
	None-smoker	58 (46.4%)	67 (53.6%)	1	1
Alcohol drinking	Drinker	90 (64.3%)	50 (35.7%)	2.03 (1.12–4.56)	1.60 (0.12–4.23)
	Non-drinker	68 (46.9%)	77 (53.1%)	1	1
Diabetic mellitus	Yes	89 (70%)	40 (30%)	2.80 (1.09–4.56)	1.32 (1.09–3.44)
	No	69 (44%)	87 (66%)	1	1
Classification of heart failure	NYHA I	30 (40%)	47 (60%)	1	1
	NYHA II	25 (45.5%)	30 (54.5%)	4.56 (1.12–6.45)	2.45 (0.89–4.77)*
	NYHA III,	40 (57%)	30 (43%)	8.45 (2.45–12.56)	3.60 (1.09–6.78)
	NYHA IV	63 (76%)	20 (24%)	12.56 (1.78–16.98)	4.67 (1.12–8.45)*
The types of medication for heart failure	Angiotensin-converting enzyme (ACE) inhibitors	10 (20.4%)	39 (79.6%)	1	1
	Angiotensin II receptor blocker	20 (57%)	15 (43%)	4.56 (1.12–68)	0.98 (0.12–4.78)
	Beta-blockers	30 (59%)	21 (41%)	5.60 (1.14–8.89)	1.34 (0.45–6.44)
	Diuretics	20 (50%)	20 (50%)	4.34 (1.12–4.56)	1.40 (0.87–4.77)
	Cardiac glycosides (digoxin)	18 (51.4%)	17 (48.6%)	3.88 (1.08–5.32)	0.45 (0.12–1.13)
	Anticoagulation or antiplatelet therapy	18 (54.5%)	15 (55.5%)	2.89 (1.18–4.67)	0.63 (0.09–4.89)

Hosmer–Lemeshow test = 0.66

BMI: body mass index

*p ≤ 0.05

## Discussion

The objective of this study was to determine the prevalence of sexual dysfunction and its associated factors among patients with heart failure in Gondar City, as well as in the public hospitals of the north and west zones of Gondar.

This study revealed that the overall prevalence of sexual dysfunction among heart failure patients was 57.92% (95% CI 54.76–63.76%). This aligns with a similar study in Cameroon, which reported a prevalence of 57.7% [40]. However, this finding is lower than that of a study conducted by Westlakeet al. who reported a significant decrease in sexual function among 75% of heart failure patients [14], and a study by Schwarz et al., which found that 84% of heart failure patients had sexual dysfunction [10]. The high prevalence of sexual dysfunction in patients with chronic heart failure can be attributed to several factors, including psychological stress, hemodynamic alterations, and comorbidities. Anxiety and depression resulting from chronic illness, changes in blood flow and pressure due to heart failure or its medications, and the presence of other chronic conditions contribute to this issue [10, 41].

In this study, female participants had a higher prevalence of sexual dysfunction compared to male participants. These findings are consistent with a study conducted in Australia [9]. Hormonal fluctuations, particularly during menopause, pregnancy, and menstruation, can significantly impact sexual function in women, leading to reduced libido, vaginal dryness, and discomfort during intercourse [42, 43].

Among the 285 male study participants, 158 (55.4%) were found to have erectile dysfunction. In this study, the prevalence of erectile dysfunction among male heart failure patients was lower than the 90% reported in a study conducted in Poland [44], and the 74% reported by Mary Medina et al. [45]. This disparity may be attributed to several factors. First, variations in access to healthcare services, including sexual health services, can influence diagnosis and treatment, leading to differences in reported prevalence rates. Additionally, differences in study methodologies, such as sample size, population characteristics, and diagnostic criteria, can significantly affect prevalence rates. Moreover, the prevalence of comorbid conditions like diabetes, hypertension, and depression, which are known to impact erectile function, may vary by country and population [46–48]. Finally, lifestyle factors, including diet, physical activity, smoking, and alcohol consumption, can also contribute to the differences in erectile dysfunction prevalence across populations [47].

In this study, a substantial proportion of female heart failure patients reported experiencing sexual dysfunction. Specifically, 63% of the 138 women who participated indicated challenges related to sexual function. However, this prevalence is lower compared to a study conducted in Australia, where 25–76% of women with heart failure reported sexual problems or concerns [9].

This study revealed sexual dysfunction in the female were significantly associate with BMI, age > 60 years, insufficient physical activity, and smoking. Sexual dysfunction was more common in female participants above the age of 60 years than in those between the ages of 25 and 40. This result is consistent with the findings from Korea, Germany, and a global study conducted in 29 countries, Egypt, and Nigeria, which showed that older age clients were significantly more likely to develop sexual dysfunction compared with the age group 40–49 years [49–53]. This could be because older age is often linked to higher rates of sexual dysfunction in patients with heart failure due to physiological changes like hormonal shifts and decreased vascular health, as well as psychological factors such as increased stress and depression. Younger individuals with heart failure may also experience sexual dysfunction, but it is typically influenced more by the condition itself, medications, or coping with illness at a younger age.

Overall, age plays a significant role, but a combination of physiological and psychological factors contributes to sexual dysfunction in heart failure patients [54–56]. The odds of having sexual dysfunction were higher among overweight or obese female heart failure patients compared to their counterparts. This study is consistent with a study conducted in China [57]. This association could be attributed to overweight or obesity, which frequently leads to endothelial dysfunction, hormonal imbalances, reduced blood flow to the genitals, and other factors contributing to sexual dysfunction. Moreover, obesity-related conditions, such as diabetes and hypertension, further elevate the risk of sexual dysfunction [58–60].

Similarly, Patients with heart failure who smoke cigarettes are more likely to experience sexual dysfunction compared to non-smokers. This finding aligns with studies from Korea, Germany, and Italy, which also indicated a higher risk of sexual dysfunction among smokers [49, 61, 62]. Smoking contributes to sexual dysfunction through several mechanisms: it damages blood vessels, reducing genital blood flow and affecting erections; it causes hormonal imbalances, particularly in testosterone; and it exacerbates psychological issues such as stress. Additionally, smoking is associated with health conditions like cardiovascular disease and diabetes, further compounding sexual problems. Overall, the combination of vascular damage, hormonal changes, psychological stress, and related health issues intensifies sexual dysfunction in heart failure patients who smoke [63–66].

Participants in the study who engaged in insufficient physical activity were found to have a higher likelihood of experiencing sexual dysfunction compared to those who maintained sufficient levels of physical activity. These findings are consistent with a study reported by Maria Cristina et al., which also demonstrated a similar association between physical inactivity and increased risk of sexual dysfunction [54]. Physical inactivity can worsen heart function, leading to poor blood circulation. Sexual function, especially in men, relies on adequate blood flow to the genital area. Heart failure already impairs circulation, and lack of physical activity further exacerbates this, increasing the risk of sexual dysfunction [67, 68].

The current study also found that erectile dysfunction among male hear failures were statistically significant with diabetic mellitus, age, classified as NYHA IV and NYHA and physical activity. Heart failure patients with diabetes mellitus were found to have sexual dysfunction more frequently than those without diabetes mellitus. This finding is consistent with a study conducted in Gondar, Ethiopia [69]. This could be attributed to the fact that among diabetic patients, as age increases, there is a heightened risk of developing peripheral neuropathy, hypertension, and impotence, which may explain the increased likelihood of sexual dysfunction [70]. Finally, participants with NYHA class III and IV heart failure showed a heightened likelihood of developing sexual dysfunction compared with those with NYHA class I heart failure. This finding aligns with a study conducted in Italy [54]. The increased risk of NYHA class III and IV heart failure could be attributed to more severe limitations in physical activity and greater symptoms, even at rest, indicating more advanced stages of heart failure. These limitations can directly impact sexual function by reducing physical stamina, increasing fatigue, and limiting overall activity levels, all of which contribute to sexual dysfunction. In addition, individuals with NYHA class III and IV heart failure may have more extensive cardiovascular complications and comorbidities, further intensifying the risk of sexual dysfunction [54, 71].

## Limitations of the study

This study has several limitations due to its cross-sectional design, which inhibits the ability to establish definitive cause-and-effect relationships. Recall bias may also be an issue, given the nature of the study. Additionally, potential bias could stem from the involvement of nurses as data collectors. Furthermore, biases related to medications and treatments for cardiovascular diseases (CVDs) could influence sexual dysfunction. Pharmacological interventions, such as antihypertensive and antidepressants, may confound the results by affecting sexual function independently of heart failure. These factors should be taken into account when interpreting the findings, as they may restrict the generalizability of the results concerning the prevalence of sexual dysfunction within this population.

## Conclusion and recommendations

This study identified a high prevalence of sexual dysfunction, with females being more affected than males. Additionally, the research identified several factors influencing sexual dysfunction among patients with heart failure, including BMI, age, cigarette smoking, diabetes mellitus, and the classification of heart failure. The study recommends that healthcare providers and other stakeholders take proactive measures to alleviate the burden of sexual dysfunction in patients with heart failure. Strategies should focus on controlling the severity of heart failure symptoms, effectively managing comorbidities, and addressing factors such as body weight, psychological well-being, and behavioral patterns. By targeting these areas, healthcare providers can work toward minimizing the risk of sexual dysfunction and improving the overall quality of life for patients with heart failure.

## Data Availability

The datasets used and/or analysed during this study are available from the corresponding author and provided upon reasonable request.

## Abbreviations

AOR: Adjusted odds ratio
CI: Confidence interval
COR: Crude odds ratio
SD: Standard deviation
